# Therapeutic Duel of Rifaximin Versus Lactulose in Hepatic Encephalopathy: A Systematic Review

**DOI:** 10.7759/cureus.86193

**Published:** 2025-06-17

**Authors:** David O Oriko, Zainab Khawaj, Muhammad Usairam Cheema, Anjali Talreja, Muhammad Abbas Tayyab, Muhammad Hamza Zamir, Maheen Iqbal, Umer Farooq, Osatohanmwen Ekomwereren, Muhammad M Tariq, Abdul Haseeb Hasan

**Affiliations:** 1 Neurology, University of Maryland Medical Center, Baltimore, USA; 2 Medicine and Surgery, Khyber Medical University, Peshawar, PAK; 3 Internal Medicine, Services Institute of Medical Sciences, Lahore, PAK; 4 Medicine and Surgery, Isra University, Hyderabad, PAK; 5 Medicine and Surgery, King Edward Medical University, Lahore, PAK; 6 Internal Medicine, Fauji Foundation Hospital, Rawalpindi, Rawalpindi, PAK; 7 Acute Medicine, Peterborough City Hospital, Peterborough, GBR; 8 Trauma and Orthopaedics, Shrewsbury and Telford Hospital National Health Service (NHS) Trust, Shrewsbury, GBR; 9 Internal Medicine, Foundation University Medical College, Islamabad, PAK; 10 Medicine, Mayo Hospital, Lahore, PAK

**Keywords:** cirrhosis, hepatic encephalopathy, lactulose, neurocognitive impairment, randomized controlled trial, rifaximin, treatment efficacy

## Abstract

This systematic review aimed to compare the clinical efficacy of rifaximin versus lactulose in the management of hepatic encephalopathy (HE) by analyzing evidence from randomized controlled trials (RCTs). A comprehensive search across major databases identified seven eligible RCTs encompassing 693 adult patients diagnosed with overt or minimal HE. Findings demonstrated that rifaximin is at least as effective as lactulose in reversing HE symptoms, with some studies reporting significantly higher HE reversal rates when rifaximin was used in combination with lactulose (e.g., 76% vs. 50.8%, *p*<0.004), reduced mortality (23.8% vs. 49.1%, *p*<0.05), and shorter hospital stays (5.8 vs. 8.2 days, *p*=0.001). While other trials reported similar efficacy between the two agents (e.g., HE improvement: 84.4% vs. 95.4%, *p*=0.315), rifaximin was generally associated with better tolerability and fewer gastrointestinal side effects. These results support rifaximin as an effective and well-tolerated therapeutic option, either as monotherapy or in combination with lactulose. Further large-scale, multicenter trials are warranted to assess long-term outcomes, recurrence rates, and cost-effectiveness.

## Introduction and background

Hepatic encephalopathy (HE) is a complex neuropsychiatric syndrome arising from liver dysfunction, commonly observed in patients with cirrhosis or portosystemic shunting [1]. It manifests with a broad spectrum of cognitive and motor impairments, ranging from subclinical alterations in attention and coordination to overt coma. The pathophysiology of HE is multifactorial, with elevated blood ammonia levels and alterations in gut microbiota playing central roles in neurotoxicity [2]. HE not only contributes to poor quality of life but also increases the frequency of hospitalisations, healthcare costs, and mortality among patients with chronic liver disease. Given its profound impact, timely and effective therapeutic strategies are crucial to prevent both initial and recurrent episodes of HE [3].

Lactulose, a non-absorbable disaccharide, has long been the cornerstone of HE management. It works by acidifying the gut lumen, promoting the conversion of ammonia to non-absorbable ammonium, and acting as a laxative to expedite ammonia excretion [4]. Despite its widespread use, lactulose is associated with gastrointestinal side effects such as bloating, diarrhoea, and non-compliance, which may hinder its long-term utility. In recent years, rifaximin, a minimally absorbed oral antibiotic, has gained attention as a novel therapeutic option. It modulates gut microbiota by reducing ammonia-producing bacteria and has demonstrated efficacy in both primary and secondary prophylaxis of HE, with a more favourable side effect profile [5]. The 2014 guidelines from the American Association for the Study of Liver Diseases (AASLD) recommend lactulose as first-line therapy and rifaximin as an effective adjunct to reduce recurrence in patients with a history of overt HE [5].

However, accessibility and cost considerations often limit the widespread use of rifaximin in many low- and middle-income countries, where lactulose remains the more affordable and readily available option. These economic factors may influence treatment decisions despite the clinical advantages of rifaximin. Given the emerging clinical data and the need for evidence-based therapeutic decision-making, it is imperative to critically evaluate and compare the efficacy of rifaximin versus lactulose in the treatment of hepatic encephalopathy. This systematic review seeks to synthesise current evidence from randomised controlled trials to determine which treatment offers superior clinical outcomes, including resolution of HE episodes, prevention of recurrence, and reduction in hospitalisations. The objective of this review is structured according to the PICO framework [6], which helps to define the study’s focus: Population (patients with hepatic encephalopathy), Intervention (rifaximin), Comparator (lactulose), and Outcomes (clinical efficacy, recurrence rates, hospitalisations, and adverse effects).

## Review

Materials and methods

Search Strategy

This systematic review was conducted in accordance with the Preferred Reporting Items for Systematic Reviews and Meta-Analyses (PRISMA) guidelines [7]. A comprehensive literature search was performed across multiple electronic databases, including PubMed, Scopus, Embase, and the Cochrane Central Register of Controlled Trials (CENTRAL). The search strategy included a combination of Medical Subject Headings (MeSH) and free-text keywords such as “hepatic encephalopathy”, “rifaximin”, “lactulose”, and “randomized controlled trial”. Boolean operators (AND/OR) were used to combine terms appropriately. The search was limited to studies published in English and restricted to randomised controlled trials involving human subjects. Reference lists of included articles were also screened to identify any additional relevant studies. The database search covered studies published from January 1, 1990, to October 15, 2023, to ensure the inclusion of both early pivotal trials and more recent randomised controlled studies relevant to the comparison of rifaximin and lactulose in hepatic encephalopathy.

Eligibility Criteria

Study selection was guided by a predefined PICO framework. The population included adult patients diagnosed with hepatic encephalopathy, both overt and minimal. The intervention was rifaximin administered either as monotherapy or in combination with other agents. The comparator was lactulose given alone or with a placebo. The primary outcomes assessed were clinical reversal of HE, hospital stay duration, mortality, and recurrence rates. Only randomised controlled trials (RCTs) were considered eligible to ensure the inclusion of high-quality evidence.

Studies were included if they: (1) involved adult patients with hepatic encephalopathy, (2) directly compared rifaximin and lactulose in any form, (3) reported measurable clinical outcomes, and (4) were published in English. Trials were excluded if they were non-randomised, involved paediatric populations, lacked a clear comparator group, or if full texts were not accessible. Studies evaluating rifaximin or lactulose alongside interventions not uniformly applied across groups were also excluded to maintain consistency in comparisons.

Data Extraction

Data extraction was performed independently by two reviewers using a standardised data extraction form. Information collected included study title, year of publication, authorship, study design, sample size, population characteristics, intervention and comparator details, and primary outcomes. Discrepancies between reviewers were resolved through discussion or third-party adjudication. When necessary, study authors were contacted for clarification of missing or ambiguous data. Extracted data were organised in tabular form to facilitate comparison across studies.

Data Analysis and Synthesis

Due to variability in outcome definitions, intervention dosing, and study populations, a qualitative synthesis approach was employed rather than meta-analysis. Studies were compared based on trends in clinical outcomes such as HE reversal rates, mortality, and hospital stay duration. The direction and consistency of effect were assessed, and emphasis was placed on statistically significant findings. The Cochrane Risk of Bias 2.0 tool was used to assess methodological quality, and findings were synthesised narratively to highlight the comparative efficacy and clinical implications of rifaximin versus lactulose.

Results

Study Selection Process

**Figure 1:**
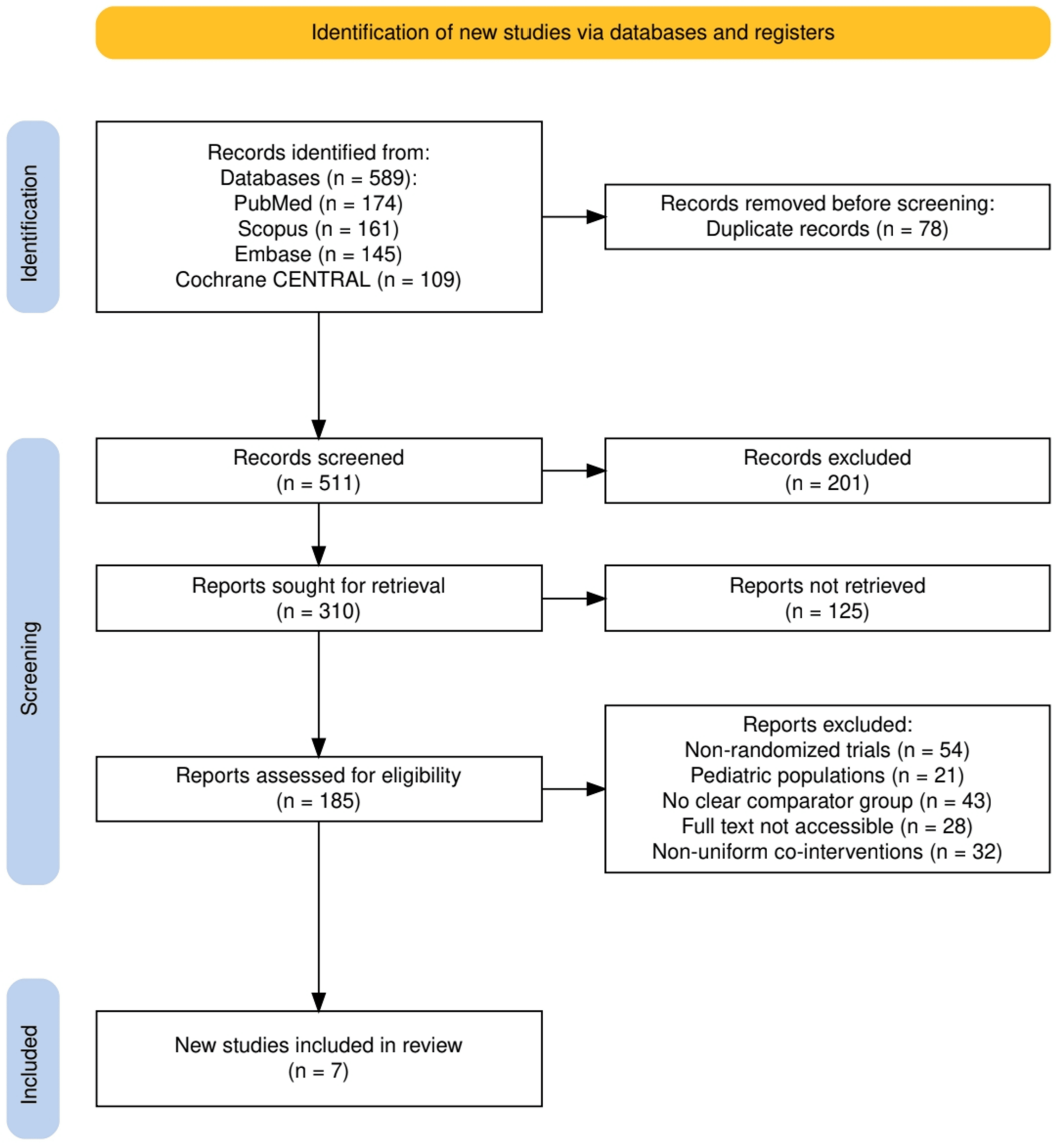
The PRISMA flowchart represents the study selection process. PRISMA: Preferred Reporting Items for Systematic Reviews and Meta-Analyses

The study selection process is illustrated in Figure 1. A total of 589 records were identified through database searches, including PubMed (174), Scopus (161), Embase (145), and Cochrane CENTRAL (109). After the removal of 78 duplicate records, 511 unique articles were screened by title and abstract. Of these, 201 records were excluded for not meeting basic relevance criteria. The remaining 310 full-text reports were sought for retrieval, of which 125 could not be accessed. A total of 185 full-text articles were assessed for eligibility. Following detailed evaluation, 178 articles were excluded for reasons including non-randomised design (54), a paediatric population (21), absence of a clear comparator group (43), inaccessible full text (28), or use of non-uniform co-interventions (32). Ultimately, seven randomised controlled trials met the inclusion criteria and were included in this systematic review.

Characteristics of the Selected Studies

The characteristics of the selected studies are summarised in Table 1. All included trials were randomised controlled trials comparing rifaximin and lactulose in patients diagnosed with hepatic encephalopathy, either overt or minimal. Sample sizes ranged from 50 to 130 participants, with balanced allocation to intervention and comparator arms. Most studies focused on patients with overt hepatic encephalopathy of varying severity, though one trial specifically targeted minimal hepatic encephalopathy (HE). The majority of participants were adults with cirrhosis, with varying Child-Turcotte-Pugh classifications [8] and model for end-stage liver disease (MELD) scores [9]. Interventions involved rifaximin either alone or in combination with lactulose, compared to lactulose monotherapy or placebo. Across studies, outcomes were measured in terms of HE reversal, mortality, hospitalisation duration, symptom improvement, and quality of life. The duration of interventions varied slightly, but all studies reported clinically relevant endpoints, allowing for a meaningful comparison of efficacy and tolerability between the two agents.

**Table 1:** The summary of the randomised controlled trials comparing Rifaximin and Lactulose in the treatment of hepatic encephalopathy. HE: Hepatic Encephalopathy, CTP: Child-Turcotte-Pugh, MELD: Model for End-Stage Liver Disease, TID: Three Times Daily, BID: Twice Daily, HRQOL: Health-Related Quality of Life, MHE: Minimal Hepatic Encephalopathy, NCT: Number Connection Test

Study (Author, Year)	Study Design	Sample Size (Rifaximin / Lactulose)	Population Characteristics	Intervention	Comparator	Outcome
Sharma et al., 2013 [10]	Randomized Controlled Trial, double-blind	63/57	120 patients with overt HE, mean age 39.4 ± 9.6 years, 89 males and 31 females; CTP class B (30.8%), C (69.2%); mean CTP score 9.7 ± 2.8; MELD 24.6 ± 4.2; HE grades 2–4	Lactulose + Rifaximin 1200 mg/day	Lactulose + placebo	Higher complete reversal of HE (76% vs. 50.8%, p<0.004), reduced mortality (23.8% vs. 49.1%, p<0.05), shorter hospital stay (5.8 vs. 8.2 days, p=0.001)
Hasan et al., 2018 [11]	Randomized Controlled Trial	48/48	96 patients with overt HE, randomized into two treatment arms	Lactulose + Rifaximin (standard dose)	Lactulose only	No significant difference in survival; lactulose-only group had higher neurological improvement and longer mean survival; authors concluded lactulose alone may be more effective
Paik et al., 2005 [12]	Randomized Controlled Trial, open-label	32/22	54 Korean patients with liver cirrhosis and hepatic encephalopathy	Rifaximin (400 mg TID for 7 days)	Lactulose (30–60 mL/day for 7 days)	Both treatments improved HE markers (blood ammonia, mental status, NCT); no statistically significant difference in efficacy (84.4% vs. 95.4%, p=0.315); rifaximin was as safe and effective as lactulose
Sidhu et al., 2016 [13]	Randomized Controlled Trial, open-label, non-inferiority	57/55	112 cirrhotic patients with minimal hepatic encephalopathy (MHE), diagnosed by ≥2 positive neuropsychometric tests	Rifaximin 400 mg TID	Lactulose 30–120 mL/day	MHE reversal rates were 73.7% (rifaximin) vs. 69.1% (lactulose); non-inferiority not established (CI crossed pre-specified margin); HRQOL improved in both groups; lactulose group had more flatulence and diarrhea
Wahib et al., 2014 [14]	Randomized Controlled Trial	25/25	50 patients with grade 1–3 HE per West Haven criteria; all received enemas and dietary protein restriction	Rifaximin 1200 mg/day for 7 days	Lactulose 90 mL/day for 7 days	Rifaximin significantly improved HE parameters (mental status, behavior, asterixis, serum ammonia); associated with shorter and less frequent hospitalization; better tolerated than lactulose
Butt et al., 2018 [15]	Randomized Controlled Trial	65/65	130 patients with grades II–IV HE due to decompensated chronic liver disease; mean age 56.1 ± 11.2 years; 53.1% male	Lactulose 30 mL TID + Rifaximin 550 mg BID	Lactulose 30 mL TID	No statistically significant difference in HE reversal after 10 days (67.7% vs. 58.5%, p=0.276); concluded similar efficacy of both regimens
Bucci et al., 1993 [16]	Randomized Controlled Trial, double-blind, double-dummy	29/29	58 patients (mean age 57 years) with cirrhosis and medium to severe portosystemic encephalopathy	Rifaximin 1200 mg/day for 15 days	Lactulose 30 g/day for 15 days	Both groups showed significant improvement in HE symptoms and serum ammonia; rifaximin had quicker effect and was better tolerated with fewer side effects

Quality Assessment

The methodological quality of the included studies was evaluated using the Cochrane Risk of Bias 2.0 tool [17], and the results are detailed in Table 2. Of the seven randomised controlled trials included, five were assessed as having a low overall risk of bias, reflecting robust trial design and adequate reporting across all evaluated domains. These studies demonstrated appropriate randomisation procedures, minimal deviations from intended interventions, complete outcome data, valid outcome measurements, and no evidence of selective reporting. Two studies were rated as having some concerns: one due to limited clarity in the randomisation process and outcome reporting, and another due to an open-label design and partial concerns regarding outcome measurement. Overall, the quality of evidence was high, supporting the reliability of the conclusions drawn in this review.

**Table 2:** The risk of bias assessment of included randomised controlled trials using the Cochrane RoB 2.0 tool. RoB: Risk of Bias, RCT: Randomised Controlled Trial

Study (Author, Year)	Randomization Process	Deviations from Intended Interventions	Missing Outcome Data	Outcome Measurement	Selective Reporting	Overall RoB
Sharma et al., 2013 [10]	Low risk	Low risk	Low risk	Low risk	Low risk	Low risk
Hasan et al., 2018 [11]	Some concerns (randomization and outcome clarity limited)	Low risk	Some concerns	Some concerns	Some concerns	Some concerns
Paik et al., 2005 [12]	Some concerns (open-label)	Low risk	Low risk	Some concerns	Low risk	Some concerns
Sidhu et al., 2016 [13]	Low risk	Low risk	Low risk	Low risk	Low risk	Low risk
Wahib et al., 2014 [14]	Low risk	Low risk	Low risk	Low risk	Low risk	Low risk
Butt et al., 2018 [15]	Low risk	Low risk	Low risk	Low risk	Low risk	Low risk
Bucci et al., 1993 [16]	Low risk	Low risk	Low risk	Low risk	Low risk	Low risk

Discussion

The comparative analysis of seven randomised controlled trials evaluating rifaximin versus lactulose in the management of hepatic encephalopathy (HE) reveals a consistent trend of comparable efficacy between the two agents, with rifaximin often demonstrating advantages in tolerability and hospitalisation outcomes. In a pivotal double-blind RCT by Sharma et al. [10], combination therapy with rifaximin and lactulose resulted in significantly higher complete HE reversal rates (76% vs. 50.8%, p<0.004), reduced mortality (23.8% vs. 49.1%, p<0.05), and a shorter hospital stay (5.8 vs. 8.2 days, p=0.001) compared to lactulose alone. Conversely, Hasan et al. [11] found no statistically significant difference in survival or neurological improvement, suggesting comparable efficacy with a slight advantage for lactulose in their cohort [18]. Paik et al. [12] and Bucci et al. [16] both concluded that rifaximin was as effective as lactulose, with Bucci et al. [16] noting faster symptom improvement and better tolerability with rifaximin. In patients with minimal HE, Sidhu et al. [13] reported similar reversal rates for rifaximin (73.7%) and lactulose (69.1%), although non-inferiority of rifaximin was not formally established. Wahib et al. [14] supported the efficacy of rifaximin in improving clinical and biochemical HE markers, highlighting its favourable hospitalisation profile. Meanwhile, Butt et al. [15] also observed no significant difference in reversal rates (67.7% vs. 58.5%, P=0.276) between rifaximin-lactulose combination therapy and lactulose alone. Collectively, these findings suggest that rifaximin is at least as effective as lactulose in reversing HE and may offer additional clinical benefits such as faster recovery, fewer side effects, and potentially reduced hospital burden, particularly when used as part of a combination regimen.

Our findings are consistent with the 2024 AASLD [19] and 2022 European Association for the Study of the Liver (EASL) guidelines [20], which endorse lactulose as first-line therapy and rifaximin as an effective adjunct to reduce recurrence and hospitalisation in hepatic encephalopathy. Landmark trials, including the pivotal study by Bass et al. [21], demonstrated that rifaximin significantly reduces HE recurrence when used in combination with lactulose. While our review corroborates rifaximin’s clinical value, especially in combination therapy, it adds nuance by highlighting that monotherapy with rifaximin may offer comparable efficacy to lactulose in certain populations, though the evidence remains mixed. These findings align with current treatment algorithms and support the consideration of rifaximin as either an adjunct or alternative based on individual patient tolerance and healthcare resource availability.

This review is methodologically rigorous, including only randomised controlled trials to ensure high-quality evidence. The study followed PRISMA guidelines for systematic review conduct and reporting. A structured Patient, Intervention, Comparison, and Outcome (PICO) framework was used to define eligibility criteria and focus the research question. Clinical outcomes were chosen for direct relevance to patient care, including HE reversal, mortality, and hospital stay. The risk of bias was assessed using the Cochrane RoB 2 tool, ensuring a transparent evaluation of internal validity across all included studies.

Several included trials had small sample sizes, limiting statistical power. Some studies were open-label, introducing potential performance bias. Short follow-up durations prevented the assessment of long-term outcomes such as recurrence or mortality. Dosing protocols for rifaximin and lactulose varied, and outcome definitions were not always standardised. The geographic concentration of studies, particularly from South Asia, limits generalisability. Additionally, heterogeneity in the use of HE grading systems and neuropsychometric tools may have influenced outcome comparability.

This review was limited by the exclusion of non-English language studies, potentially introducing language bias. Some full-text articles were unavailable, possibly omitting relevant data. A quantitative meta-analysis was not performed due to clinical and methodological heterogeneity. There is also potential for publication bias, as studies with negative or non-significant findings may be under-represented. Lastly, only trials published within a certain timeframe were included, which may have excluded older but still relevant data.

Our findings suggest that rifaximin is a clinically effective and well-tolerated alternative or adjunct to lactulose in the management of hepatic encephalopathy [22]. While combination therapy appears superior in reducing mortality and hospital stay, monotherapy with rifaximin may be appropriate for patients intolerant to lactulose [23]. In resource-limited settings, cost considerations may favour lactulose, but the superior tolerability of rifaximin supports its use in recurrent or refractory cases where adherence is critical [24].

Future research should include large-scale, multicenter RCTs directly comparing rifaximin and lactulose monotherapy with long-term follow-up to assess recurrence, survival, and quality of life [25]. Cost-effectiveness analyses are needed to guide decision-making in various healthcare settings. Subgroup studies are warranted to explore differential outcomes in populations such as minimal HE, post-transjugular intrahepatic portosystemic shunt (TIPS) patients, and those with different aetiologies of liver disease. Standardised outcome measures and HE grading should be uniformly applied to improve data synthesis in future reviews.

## Conclusions

This systematic review demonstrates that rifaximin is at least as effective as lactulose in the management of hepatic encephalopathy and may offer added benefits in terms of tolerability, hospitalisation reduction, and patient adherence. Combination therapy appears superior in improving clinical outcomes, but monotherapy with rifaximin remains a viable alternative, especially in patients intolerant to lactulose. These findings reinforce current guidelines while supporting more flexible, patient-centred treatment strategies. Until more robust head-to-head trials with standardised outcomes are available, clinicians may consider rifaximin, either as monotherapy or in combination with lactulose, based on individual patient profiles, adherence potential, and resource availability. Future large-scale trials are essential to refine long-term efficacy, cost-effectiveness, and the optimal positioning of rifaximin in HE management.
